# Early South Americans Cranial Morphological Variation and the Origin of American Biological Diversity

**DOI:** 10.1371/journal.pone.0138090

**Published:** 2015-10-14

**Authors:** Mark Hubbe, André Strauss, Alex Hubbe, Walter A. Neves

**Affiliations:** 1 Department of Anthropology, The Ohio State University, Columbus, Ohio, United States of America; 2 Instituto de Investigaciones Arqueológicas y Museo, Universidad Católica del Norte, San Pedro de Atacama, Región de Antofagasta, Chile; 3 Department of Human Evolution, Max Planck Institute for Evolutionary Anthropology, Leipzig, Germany; 4 Departamento de Oceanografia, Instituto de Geociências, Universidade Federal da Bahia, Salvador, Bahia, Brazil; 5 Laboratório de Estudos Evolutivos Humanos, Departamento de Genética e Biologia Evolutiva, Instituto de Biociências, Universidade de São Paulo, São Paulo, Brazil; University of Otago, NEW ZEALAND

## Abstract

Recent South Americans have been described as presenting high regional cranial morphological diversity when compared to other regions of the world. This high diversity is in accordance with linguistic and some of the molecular data currently available for the continent, but the origin of this diversity has not been satisfactorily explained yet. Here we explore if this high morphological variation was already present among early groups in South America, in order to refine our knowledge about the timing and origins of the modern morphological diversity. Between-group (Fst estimates) and within-group variances (trace of within-group covariance matrix) of the only two early American population samples available to date (Lagoa Santa and Sabana de Bogotá) were estimated based on linear craniometric measurements and compared to modern human cranial series representing six regions of the world, including the Americas. The results show that early Americans present moderate within-group diversity, falling well within the range of modern human groups, despite representing almost three thousand years of human occupation. The between-group variance apportionment is very low between early Americans, but is high among recent South American groups, who show values similar to the ones observed on a global scale. Although limited to only two early South American series, these results suggest that the high morphological diversity of native South Americans was not present among the first human groups arriving in the continent and must have originated during the Middle Holocene, possibly due to the arrival of new morphological diversity coming from Asia during the Holocene.

## Introduction

The cranial morphological diversity of native American groups over time has been an important source of information about the processes of human occupation of the New World, and has often been used to support different settlement scenarios for the Americas [1–20]. Although distinct scenarios for the occupation of the New World have been supported through the study of local cranial morphological diversity, most rest on the notion that early Americans shared a distinct morphological pattern from the one seen among most late/recent native Americans. These differences suggest high diachronic morphological diversity [8], where early South Americans (and to a certain degree early North Americans [21]) differed significantly in terms of cranial morphology from their late and modern counterparts [5–6,9,16,22–23]. However, recent studies have revealed a high degree of biological variability even when only late/recent native South Americans are considered [11,14,24–27], indicating that the high morphological diversity in South America is not only restricted to differences over time.

A high morphological diversity among recent South American groups is in sharp contrast to the molecular studies that demonstrate a general loss of genetic diversity associated with increased distance from Africa [28–30], with native American populations presenting the lowest genetic variances among all continents. A similar decrease in variance with increased distance from Africa was also reported for worldwide cranial morphological diversity [31–32]. However, this loss of within-group variance, explained as the result of multiple founder effects and expansion range effects from populations migrating out of Africa, is not correlated with the degree of population structure, or differences between groups in the Americas. When the apportionment of the variation due to between-group differences is considered, South America has been described as highly diverse. Linguistic studies, for instance, demonstrate that South America is impressively diverse as far as native languages are concerned [33–34]. Nettle [34] defends the idea that high linguistic diversity is a consequence of the rapid group fission and relative isolation once people arrived in the unoccupied South American lowlands. Similarly, although South Americans present low overall within-group molecular variance, differences between groups, as measured by Fst values, of eastern South Americans has been reported to be high. Wang et al. [30] (p. 2052), for example, report Fst values for Eastern South America (14.7%) more than twice as high as Fst for series worldwide (7.1%), indicating high population structure among recent eastern native South Americans. Consequently, the high levels of cranial morphological differences between groups reported for recent South Americans is in accordance with the idea of high between-group differentiation, despite the loss of intra-group variation associated with distance from Africa.

Even though these similar patterns can be observed in South America, regarding morphological, genetic and linguistic diversity, the overall genetic diversity seen in the continent is smaller than what has been described in cranial morphological studies [28–30]. This incongruence between morphological and molecular diversity has been used in the past to criticize the use of the high morphological differences in the New World as an indicator of high biological diversity in the continent in the past [8], However, phenotypic variance may not be correlated to the genetic variance of those loci that are not influencing the phenotype, such as the loci under study in most of the molecular analyses dealing with the settlement of the Americas [35]. As such, the study of cranial morphology as an independent and informative source for the estimation of phylogenetic relationships among populations cannot be discarded, especially given that, unlike molecular and linguistic data, which are largely restrained to recent samples, cranial morphology allows us to investigate the origin of the high inter-group diversity seen in the continent by assessing the within and between-group variation of early South American samples.

Despite the large number of studies demonstrating the high cranial morphological differences between early and late native American groups, the morphological variance present among the first humans who occupied the New World has been scarcely studied (see [21] and [36] for exceptions). Powell [10], for instance, presented a scenario favoring microevolution within the New World to explain the marked differences in terms of cranial morphology between early and recent native Americans, based on the assumptions that the first Americans exhibited an especially high degree of biological diversity and that genetic drift (mainly due to group fission) acting on the highly variable parent population could explain the origin of the morphological differentiation observed among late native American populations. However, his scenario is based on the scant early material available in North America, which is entirely composed of isolated specimens and lack population parameter estimates. This is a limiting factor also confronted by other studies dealing with the early North American remains (e.g., [21]).

Sardi et al. [26] also recognized that early and recent native South Americans display very different cranial patterns. Moreover, they do not dismiss the possibility that the morphological pattern of Late Holocene populations was generated *in situ* from the early morphological pattern by means of local stochastic processes of differentiation. In their opinion, however, the local differentiation scenario would be feasible only if early South Americans displayed an uncommonly high degree of biological variance within-groups, which could then be later partitioned and structured differently between groups by genetic drift and group fissions during the Holocene. A similar scenario is also proposed by Gonzalez-José et al. [8], to accommodate both the molecular and morphological diversity observed in the continent. According to these authors, a highly morphologically diverse population was present in the early stages of the settlement of the continent, which maintained continuous gene-flow with Asia (see also [19]). Under this scenario the combination of high diversity and recurrent gene-flow with Asia could explain the high cranial morphological diversity and low molecular diversity in the continent.

Consequently, understanding if the high cranial morphological variation seen among recent native Americans was already present among the continent’s early human groups is crucial in discussing the processes of morphological diversification and human dispersion in the continent. Given the importance that the within and between-group variances of early American populations has in the recent models that try to explain the origin of the morphological diversity in the continent [8,16–17,19], it is essential to estimate these parameters to test the validity of some of the population parameter assumptions made in previous studies. Therefore, here we address this question by estimating within and between-group variances of early South Americans compared to modern human population values, and explore the consequences of this information for our understanding of the processes by which South America was settled during the late Pleistocene.

## Materials and Methods

Morphological variances within and between populations were assessed based on 23 linear craniometric measurements described by Howells (Table 1 [37,38]). Two cranial series were used to represent early morphological variability in South America (Table 2; S1 Dataset): Lagoa Santa (11.5–7.5 kyr BP) in east-central Brazil and Paleo Colombia (10.5–7.0 kyr BP), from Sabana de Bogotá, Central Colombia. The morphological affinities and archaeological context of these series have been extensively described elsewhere [6,9]. Despite spanning over three thousand years of human occupation, these two collections represent the only skeletal series in the continent with enough individuals recovered to allow the estimation of within-group parameters, and therefore offer a unique opportunity to explore the early American groups based on population estimates. All other early skeletons from the continent are represented by isolated or only a few specimens (e.g., [10, 21]).

**Table 1:** Craniometric variables used in this study.

Variables included^a^
Glabello-occipital length (GOL)
Nasio-occipital length (NOL)
Basion-bregma height (BBH)
Maximum cranial breadth (XCB)
Maximum frontal breadth (XFB)
Biauricular breadth (AUB)
Biasterionic breadth (ASB)
Nasion-prosthion height (NPH)
Nasal height (NLH)
Orbit height (OBH)
Orbit breadth (OBB)
Bijugal breadth (JUB)
Nasal breadth (NLB)
Bimaxillary breadth (ZMB)
Bifrontal breadth (FMB)
Nasio-frontal Subtense (NAS)
Biorbital breadth (EKB)
Malar length, inferior (IML)
Malar length, maximum (XML)
Cheek height (WMH)
Frontal cord (FRC)
Parietal cord (PAC)
Occipital cord (OCC)

^a^—measurement definitions according to Howells (1973, 1989).

**Table 2:** Craniometric series included in the analyses.

Population	Regional/chronological affiliation	Sample size	Males/Females ratio	% Missing values	Reference
Lagoa Santa	Early America	29	18/11	16.94	9
Paleo Colombia	Early America	14	6/8	6.52	6
Peru	South America	110	55/55	0	37, 38
Botocudo	South America	32	16/16	1.90	25
Archaic Colombia	South America	33	12/21	11.86	6
Tapera	South America	47	26/21	5.28	25
Cabeçuda	South America	19	12/7	13.50	25
Tupi-Guarani	South America	23	14/9	2.65	25
Arikara	North America	69	42/27	0	37, 38
Santa Cruz	North America	102	51/51	0	37, 38
Eskimo	North America	108	53/55	0	37, 38
North Japan	East Asia	87	55/32	0	37, 38
South Japan	East Asia	91	50/41	0	37, 38
Hainan	East Asia	83	45/38	0	37, 38
Buriat	East Asia	109	55/54	0	37, 38
Australia	Australo-Melanesia	101	52/49	0	37, 38
Tasmania	Australo-Melanesia	87	45/42	0	37, 38
Tolai	Australo-Melanesia	110	56/54	0	37, 38
Berg	Europe	109	56/53	0	37, 38
Norse	Europe	110	55/55	0	37, 38
Zalavar	Europe	98	53/45	0	37, 38
Zulu	Sub-Saharan Africa	101	55/46	0	37, 38
Dogon	Sub-Saharan Africa	99	47/52	0	37, 38
Teita	Sub-Saharan Africa	83	33/50	0	37, 38
Easter Island	Polynesia	86	49/37	0	37, 38
Mokapu	Polynesia	100	51/49	0	37, 38
Moriori	Polynesia	108	57/51	0	37, 38

Within and between-group variance apportionment of early Americans was contrasted with the values obtained for series representing recent native Americans, East Asians, Europeans, Sub-Saharan Africans, Australo-Melanesians, and Polynesians from the Howells database [39] (Table 2). These series were complemented with other native South American late Holocene and modern series, which were included in the analyses to increase the representativeness of the South American morphological variation (Table 2). Details on these series are discussed elsewhere [27]. All South American series, with the exception of Peru (measured by Howells) were measured by one of us (WAN) following the same protocol. Access to the South American remains was granted by the institutions housing them (See S1 Dataset for details). No destructive analysis was done for this study. No permits were required for this study, which complied with all relevant regulations.

Within group variance was estimated using the trace of the covariance matrix (VCV) of the series after standardizing all variables into Z-scores. VCV trace was calculated for each series independently. Since variance estimations are affected to some extent by small sample sizes, to compare the VCV trace of the early South American series with the worldwide series, random subsets with the same number of individuals as the early series were selected a thousand times from each series and the results were used to build the variance distributions for each of these. Consequently, the comparisons with Lagoa Santa were based on 1000 within-group variances calculated from subsets of 29 individuals for each series (with the exception of Cabeçuda and Tupi-Guarani, which have smaller sample sizes than Lagoa Santa. For these two series, permutations were done based on their original sample size). For the Paleo Colombian series the same number of variances was calculated from subsets of 14 individuals per series. The variances observed within the early series were plotted in a graph with the distribution of the random sets of each series to compare the results visually.

Inter-group morphological variability between regions and among series within each region was quantified by means of Fst estimates, obtained by averaging the principal diagonal of the R-matrix (r_ii_) extracted from the phenotypic data. Fst gives an estimation of the apportionment of between-group genetic variation [40–43]. Fst estimates for metric data are minimum estimates and can greatly underrepresent inter-group variation apportionment if the heritability values of the traits (measurements) are low [42,44]. Heritability values for human cranial dimension range from moderate to high [45], although different traits show very distinct heritability levels [46–47]. However, assuming mean heritability values of 0.55 in the past produced similar apportionment values to neutral molecular data [42,46], showing that even when using average heritability values, craniometric data generates comparable Fst estimates. Therefore, all Fst estimates calculated here assume a constant heritability of 0.55 to improve comparability of the results with previous studies [44–45,48].

Initially, Fst was calculated between all pairs of series, using the pooled within group VCV for all groups to calculate the R matrix [42]. The use of the pooled VCV among all groups was required because of the small sample size of some of the archaeological series, which resulted in non-reliable (i.e., weakly correlated) VCVs between groups, which therefore biased the values of Fst between pairs of groups. Fst in this case can be considered a measurement of distance, since it will reflect the distance between each group centroid to the overall centroid (i.e., if the data were not divided into groups). Still, the pairwise calculations allow exploration of the morphological affinities between series included in the study. The pairwise Fst matrix was represented graphically with a Kruskall non-metric Multidimensional Scaling (MDS [49]). To explore the confidence of the affinities observed in this case, the analysis was repeated with 100 bootstraps of the data, respecting the original sample size of the series. The bootstrapped MDS configurations were then superimposed on the original data using Procrustes analysis [50], allowing the results to be combined in a single scatterplot.

Complementing the pairwise analysis, Fst estimates and their standard errors [40–42] were also calculated for series within each of the large regions in the dataset. For the American series, Fst estimates were calculated once with all series, and then for the early series alone, for all late American series, and for all South American Late series separately, to explore the impact that diachronic changes have in the apportionment of the variation in the New World. Also, given that our series include two groups that lived in extreme cold environments (Eskimo and Buriat), which have cranial morphology responding adaptively to this environmental factor [51–52], Fst estimates were also calculated for the Americas and East Asia without these groups.

Prior to the analyses, missing values in the early American and other archaeological series (see Table 2 for details) were estimated through multiple regressions, using the overall mean of the missing variables as the dependent value and the individual’s remaining variables as independent variables (the reasoning behind this replacement has been covered elsewhere [17] and consequently we will not elaborate on it here). All analyses pooled males and females together, to maximize sample sizes of the early American series. Although pooling sexes together will inflate the within group variances, this is unavoidable in this case, since a subdivision of the prehistoric series would result in very unreliable estimates of within group variances due to low sample sizes. Even though the proportion of males and females in the series is not always similar (Table 2), sexual dimorphism should not affect the comparative results significantly, because in all series each sex still represents a significant portion of their individuals (i.e. in none of the series one of the sexes is represented by few individuals). Nonetheless, the within-group variances reported here must be considered as overestimations since they include the sexual dimorphism within series. All analyses were done in R [53], with functions written by MH, complemented by functions from packages MASS [54] and vegan [55].

## Results

**Fig 1:**
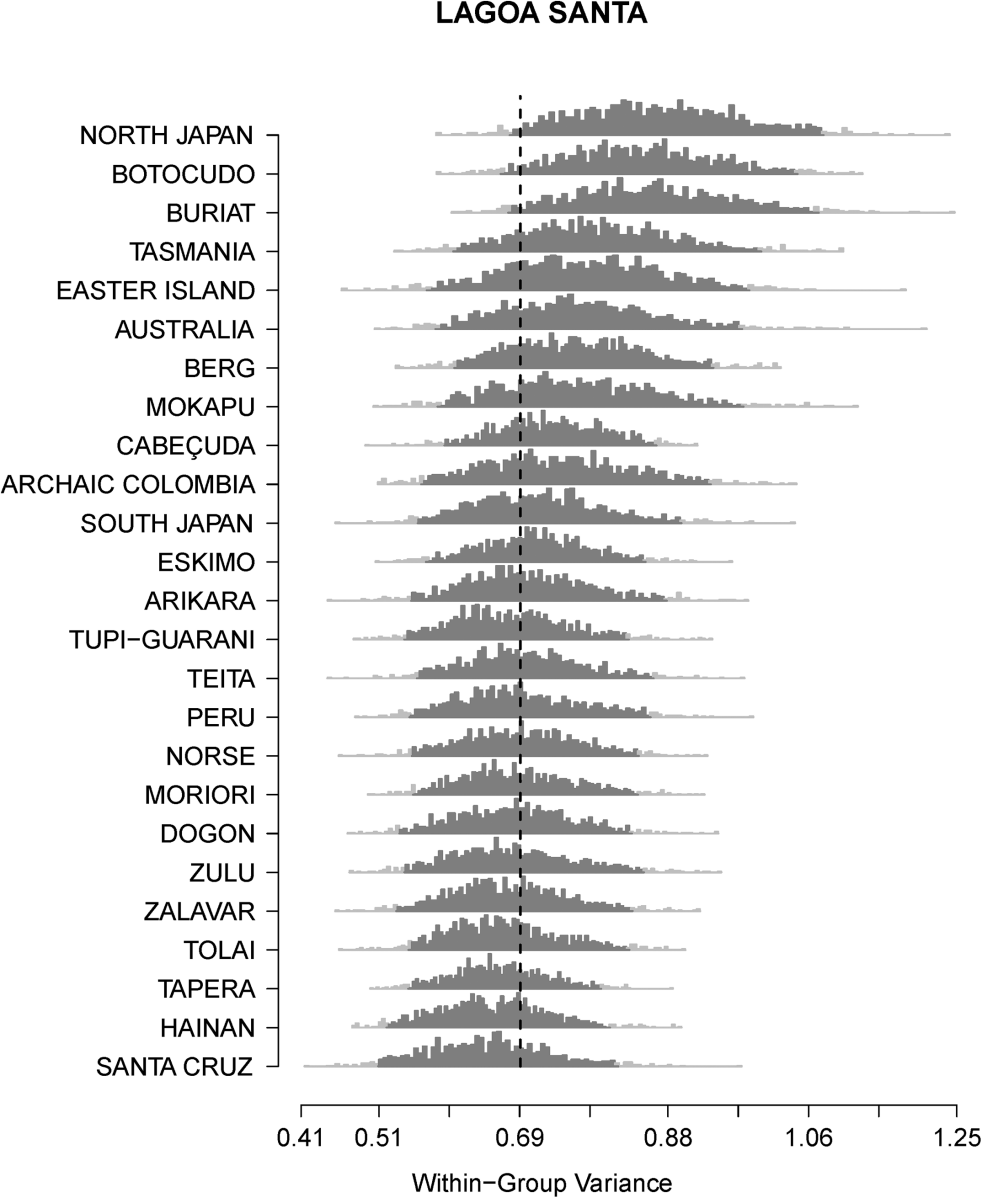
Comparison between the within-group variance of Lagoa Santa and the variance distributions generated for the reference series. The dashed line indicates the variance calculated for Lagoa Santa, and each of the grey histograms show the distribution of variances based on 1,000 random selections of 29 individuals from all reference series with larger sample sizes.

**Fig 2:**
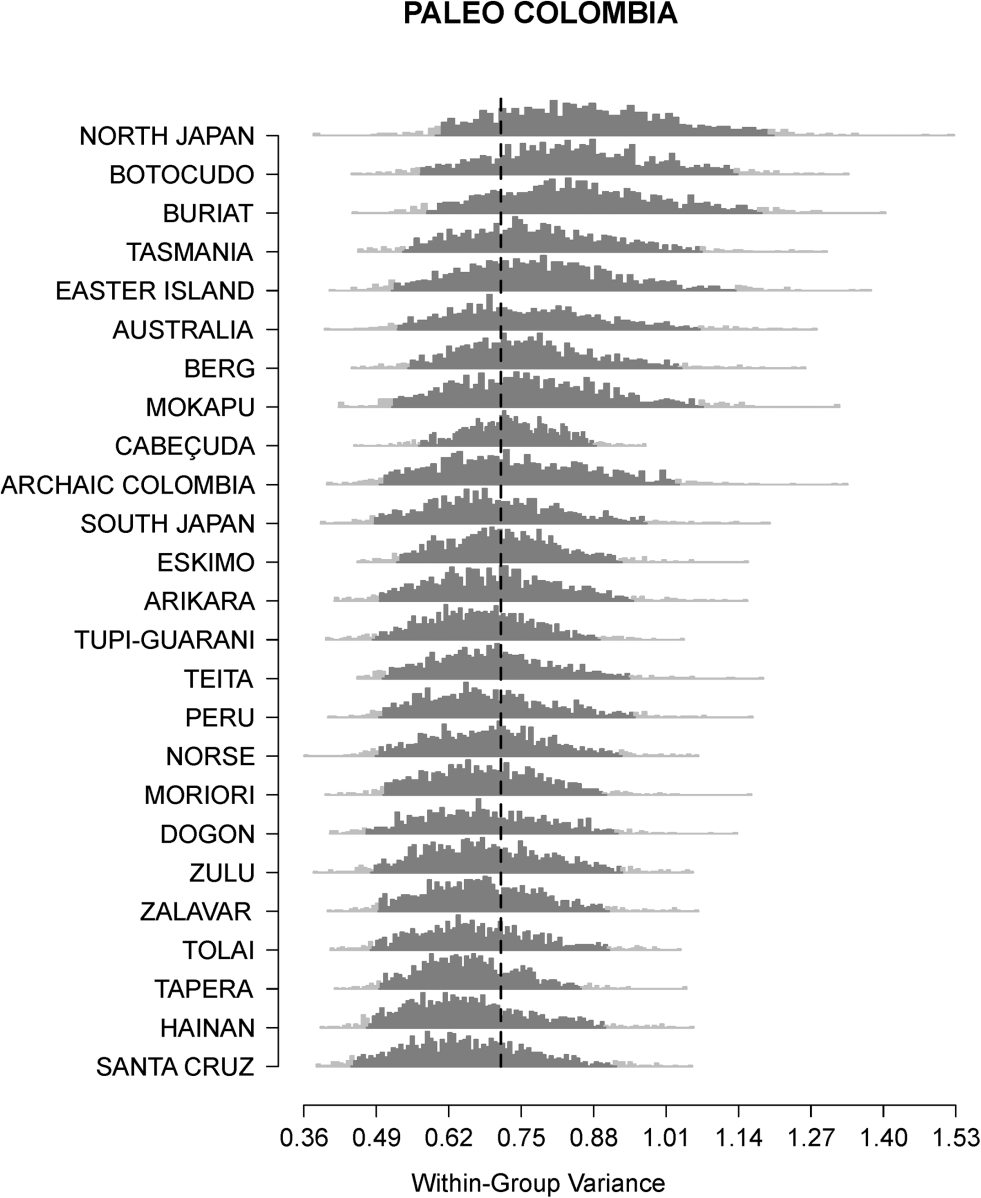
Comparison between the within-group variance of Paleo Colombia and the variance distributions generated for the reference series. The dashed line indicates the variance calculated for Paleo Colombia, and each of the grey histograms show the distribution of variances based on 1,000 random selections of 14 individuals from the reference series.

Figs 1 and 2 show the comparison between the within-group variances of Lagoa Santa and Paleo Colombia, the two early South American series included in this study, to the distributions generated from the bootstraps of the worldwide modern reference series. In both cases, the worldwide within-group variances overlap considerably, with North Japan, Botocudo and Buriat showing a slightly larger variance distribution. In the context of the reference series, both early American groups have moderate within-group variances falling inside the 95% confidence interval of the modern human series, with Lagoa Santa and Colombia presenting remarkably similar within-group variances.

Table 3 presents the Fst estimates used to assess between-group differences in the data. Fst were calculated for different combinations of the series in the study. With the exception of the Americas, all regions in the world show Fst values considerably lower than the Fst observed among series worldwide. In the Americas, the Fst observed is similar (0.24) to the worldwide one (0.28). This increased differentiation between groups is not present among early Americans, since the Fst for these groups (0.07) is closer to the Fst observed for the other regions in the world in modern times. When early Americans are removed, the Fst estimate among the American series is still high (0.24), even when Eskimos are removed (Fst = 0.23). When only South American series are considered in the analysis, the Fst estimate still is remarkably high (0.22), showing high levels of between group differentiation in the continent, corroborating previous studies [26]. These results suggest that the high morphological differences described for the American series in previous studies was not present among early groups in the continent.

**Table 3:** Fst values (h^2^ = 0.55) within regions and chronological period in the study.

Region/Chronological period	Fst	SE
World	0.276	0.002
All America	0.235	0.005
Early America	0.068	0.011
Late America	0.239	0.005
Late North America	0.205	0.006
Late North America without Eskimos	0.105	0.006
Late South America	0.224	0.007
East Asia	0.170	0.005
East Asia without Buriat	0.041	0.004
Australo-Melanesia	0.096	0.005
Sub-Saharan Africa	0.089	0.005
Europe	0.058	0.004
Polynesia	0.158	0.005

**Fig 3:**
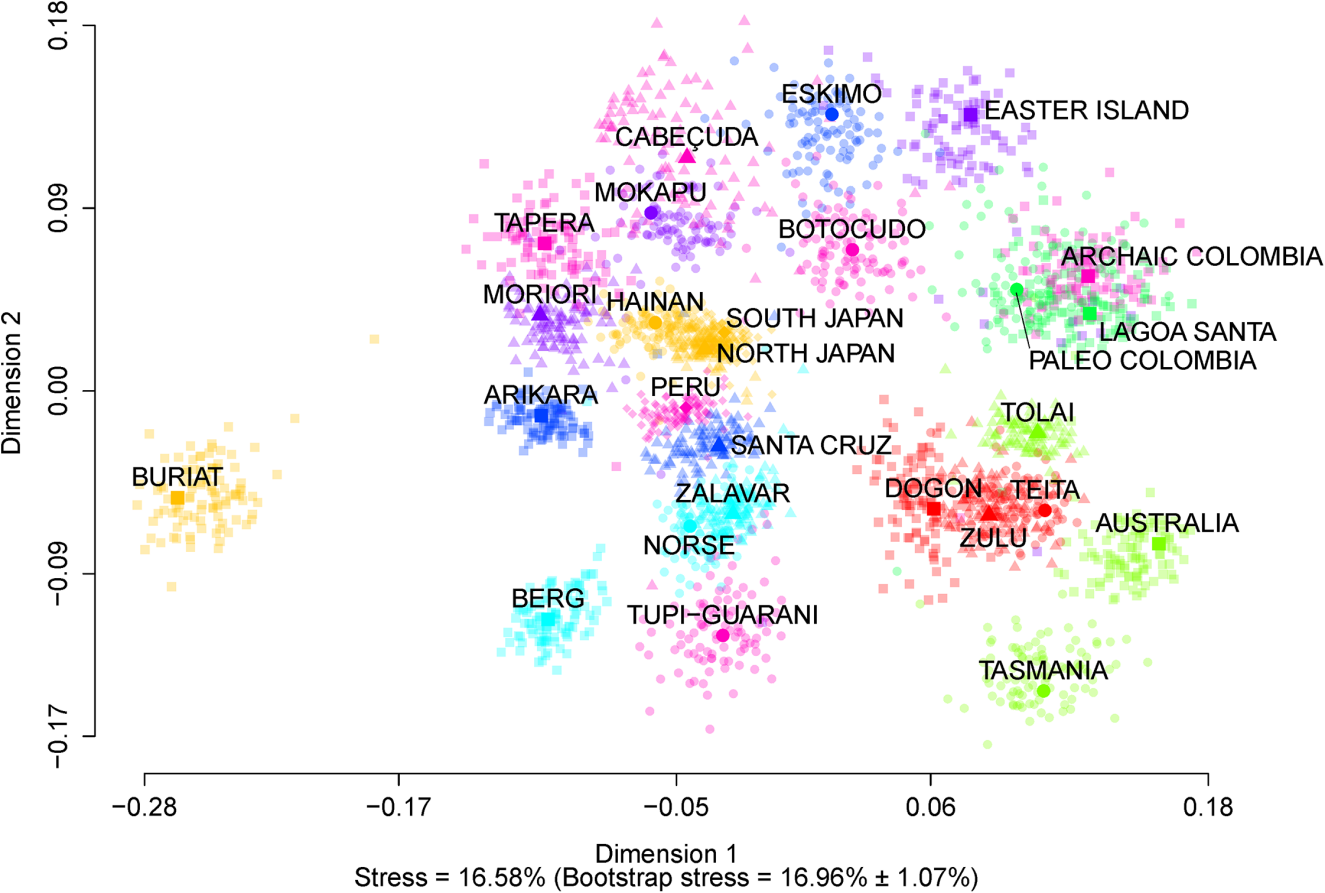
Multidimensional scaling (MDS) of the pairwise Fst matrix between series. The solid dots represent the MDS for the Fst matrix of the original data, and the transparent dots represent the MDS from bootstrapped data superimposed on the original MDS with Procrustes analysis. Series from the same region have been represented with the same colors, following the regional assignations given in Table 3.

Fig 3 shows the MDS scatterplot representing the pairwise Fst matrix between all series, which allows us to explore how the variance apportionment worldwide is distributed in terms of morphological affinities among series. The MDS plot shows that most of the diversity seen is due to differences among regions. With the exception of the Americas and Polynesia, series within most regions regions overlap when the bootstrap distribution is taken into account, with Australo-Melanesians appearing close to Sub-Saharan Africans, Europeans close to the North American series (with the exception of Eskimos), which appear in an intermediate position between the former and East Asians and some of the Polynesian series (Mokapu and Moriori). The only conspicuous outlier population in our analysis is Buriat, a NE Asian series that has been shown to have a peculiar cranial morphology probably due to the adaptive responses to extreme cold climates [51–52]. Corroborating the Fst values by region (Table 3), the differences among South American series are very marked. However, these differences are not only due to the chronology of the series, since early Americans show a great overlap among themselves and with Archaic Colombia. In other words, the differences among South American series are as high as those seen between continents (e.g., Australo-Melanesia and East Asia) in modern times.

## Discussion and Conclusions

Our results corroborate previous studies that show South America is characterized by very high levels of cranial morphological differentiation between groups [14, 24, 26–27]. They also complement previous studies by suggesting that the high cranial morphological diversity seen among late/recent native Americans was not present among the early American series investigated. The within-group variances observed for the two available early South American series was comparable to modern populations, despite the fact that each of these series represents more than three thousand years of human occupation in the continent. In other words, these results show that morphological pattern that characterized early American groups remained relatively unchanged for longs periods of time both in Central Brazil and in Colombia, since any temporal trend in cranial morphological change in these series would result in an inflated within-series variance when individuals from different time periods are pooled together. The fact that these early South Americans series show within-group populations within the range of modern population samples have important implications for the understanding of the origin of the biological diversity of native South Americans. First, these results contradict previous ideas that early Americans were highly diverse and as such could be the source, through group fission and genetic drift, of the recent morphological diversity. Second, the variance within the range of modern human samples support the use of the two early American series included here as valid units of analysis from the perspective of morphological affinities, i.e. the use of such collections as operational taxonomic units in studies of morphological affinities between series can be considered as a valid assumption based on these series’ variances.

When between-group diversity is considered the early South Americans’ Fst estimate is considerably low, falling well within the range of the other regions of the world (Europe, Australo-Melanesia, Sub-saharan Africa), especially when series that have shown strong adaptive responses to climate are removed (East Asia without Buriat, and North America without Eskimo). Indeed, the only two regions that show particularly high Fst estimates are South America and Polynesia, which has also been previously observed [56]. Polynesia is expected to show increased between-group variance apportionments due to the fact that islands have stronger natural barriers to gene-flow in the form of the ocean stretches separating them. South America, however, not only does not present the same level of natural barriers as the widely dispersed islands of Polynesia, but also shows a larger proportion of the total variance due to differences between groups than Polynesia. South America is the only continent that has between-group differences on a similar scale as when we consider all populations worldwide. Thus, South America is particularly interesting in terms of the development of modern human cranial morphological diversity, especially given that our results suggest that the high diversity seen among late native South Americans was not present among early groups entering the continent, as proposed before [8,10,19]. In other words, we argue that the high morphological diversity seen in South America today must have been generated and/or arrived after the Pleistocene/Holocene transition, long after the arrival of the first humans on the continent.

Recently, Hubbe et al. [17] suggested, based on analyses of cranial morphological affinities, that the morphological pattern seen among early South Americans is a retention of the morphological pattern that characterized other human groups by the end of the Pleistocene in the Old World (specifically in Europe and East Asia). Populations worldwide retained a similar morphological pattern throughout most of the modern human dispersion across the World, and fast changes occurred during the end of the Pleistocene and across the Holocene, especially in Europe and Asia [57–59], and consequently changes observed in South America can then be seen as an extension of what happened in the rest of the world. However, the morphological diversity observed in South America is different from the one observed elsewhere in two aspects: first, the transition from the Paleoamerican morphology to the modern morphological diversity seems to have occurred faster in South America than in the other regions. To date there is no evidence of changes in the overall cranial morphological pattern or in its variance before 7.5 kyr BP [2–6], and our results strongly support this since both our early American series do not show strong morphological differences between groups (Table 3; Fig 3) nor increased within-sample variance in relation to the other groups (Figs 1 and 2), despite representing over three thousand years of human occupation in each region. Second, in the regions where a strong morphological differentiation process is observed in the Old World (e.g., Europe and East Asia), the modern populations included in our analyses do not show strong differences between group (i.e., the regional Fst estimates are low), whereas South America presents a different pattern, where there is an extreme increase of morphological differences between groups by the end of the Holocene, some of them retaining a similar morphological pattern as the early Americans (e.g. the Archaic Colombia series [6], included in this study, the Pericu Indians from Baja California [60] and the Botocudo Indians to some extent [61]), some of them diverging considerably from the early Americans (e.g., Peru and the coastal shellmound series). Yet, this has to be viewed with caution at the moment, since our analyses only include a few series from each of the larger geographic regions explored here and it is possible that they are underrepresenting the local morphological diversity in these regions. Nonetheless, South America shows as much between-group variance apportionment as seen worldwide, indicating that several different morphological patterns were present and sometimes coexisted in the continent during the Holocene, even if the values observed for the reference regions are underestimated.

However, the reiteration that South America has high levels of morphological differences between groups (see also [26] and [56]) does not contribute necessarily to our understanding of how this morphological variation originated. The fact that the high morphological diversity observed in South American modern populations was not present among the two early South American series included here, must have been a result of two distinct (and complementary) kinds of processes, namely strong *in situ* microevolutionary processes (by random and non-random forces [10,14]) or the migration of populations carrying new morphological diversity into the continent after South America’s initial settlement [6,8–9,16,19].

Theoretically, the idea of microevolutionary processes promoting *in situ* morphological differentiation can be endorsed by our knowledge that the human skull, as the skull of many if not all mammals, is organized in development/functional modules [62–66], which can be defined as sets of highly intercorrelated traits that are less correlated with other such sets [67–71]. Modularity may enhance evolvability [71], which is the capacity of a given population to evolve [72–73], since modules allow the underlying genetic architecture to interact with selection to produce an evolutionary response [74]. When compared to the modular organization of other mammals, ours is one of the most flexible to respond in the same direction of natural selection [65,75], due to the fact that our modules are less strongly integrated with other modules in the skull. The increased evolvability of human skulls is not only restricted to selection. Among mammals, *H*. *sapiens* has a high number of dimensions in the morphospace defined by the genetic covariance matrix, which allows drift to accumulate morphological changes in many potential directions [63–64,74].

Given this background, it is hard to test the relative contribution of different evolutionary forces on the origin of the morphological diversity seen in the continent, and studies that addressed this problem have generated conflicting results [16,19]. However, we argue that *in situ* processes (guided by natural selection or genetic drift) can be tentatively excluded as a strong component of the morphological differentiation in South America and that the entrance of extra-continental morphological diversity (either through discrete dispersals or recurrent gene-flow) is a more parsimonious explanation given our current knowledge of modern human morphological variation.

Morphological changes associated to adaptive responses to climate [51–52] and life-style change [14,76] have been observed among modern humans, although these seem to be localized to specific anatomical regions or restricted to populations inhabiting extremely cold environments. However, South America does not present the extreme climatic range to explain the morphological diversity seen in the continent as a result of adaptation to cold climate, and although some of the changes observed in the continent are correlated with the adoption of agriculture [14, 76], contradictory evidence in this regard exists when the continent is seen as a whole. In Brazil for example [61] the shellmound populations show strong departure from the morphological pattern that characterizes early Americans (see also Fig 3), despite maintaining a fisher-hunter-gatherer life style.

Neutral evolutionary processes resulting from genetic drift and strong and long-lasting gene-flow barriers in the continent are also hard to sustain at this moment as reasons to explain the origin of the morphological diversity in the continent. First, the time for such a large amount of changes to be developed in South America seems to be too short if we consider the time needed for comparable changes in other parts of the world. Our results suggest early Americans did not present uniquely high within-group variances and very low differentiation between regions and across time, implying that during the Holocene a similar degree of morphological differentiation between groups appeared as the one that occurred between continents worldwide in a much longer period of time (see Fst estimates in Table 3). Second, if adaptive forces have indeed not played a major role in the morphological differentiation of South American groups, it would be expected that genetic drift and the multiple founder effects associated with range expansions from the parent populations (early Americans) would generate loss of variability across time, similar to what is seen associated with distance from Africa [32–33,77–78]. Although our study is limited to a few South American series, only two of these representative of early Holocene groups, our results do not favor a loss of variability between early and late groups, but rather the opposite. In sum, there is no strong evidence to favor that South America had unique characteristics to allow fast morphological evolution either by random or non-random forces.

Consequently, unless new evidence appears in the future refuting our current understanding of how modern human cranial morphological diversity evolved, it is hard to defend exclusively local processes as responsible for the unique level of morphological differentiation seen between groups in South America. Therefore, our results would favor a scenario where additional diversity arrived in the continent after its first occupation, either through discrete waves of human dispersion into the continent [9] or through a constant or semi-constant gene-flow with outside regions [8,19] (see also Ray et al.[79] for molecular data suggesting a similar scenario). Evidently, our results at present are limited by the few South American samples available in this study and the formal testing of this hypothesis will demand the inclusion of more series in the future.

Although the notion of external diversity influx into the continent during the Holocene has not found support in most of the molecular studies concerning native American biological diversity conducted in the past decade [30, 80–82] (but see Reich et al. [83] for a more complex scenario), recent studies based on rare alleles have suggested that a single dispersion wave might not be enough to explain their presence in the continent [84–85]. As such, the molecular data available to date do not eliminate the possibility of external diversity influx into the continent during the Holocene.

In conclusion, despite being limited to the analysis of only two early South American series, the results presented here contribute to our growing knowledge about the origins of the biological diversity of native American groups during the Holocene, by showing significant differences in the apportionment of variation across time in the continent. Under this scenario, the biological diversity that characterizes South American populations originated only during the Holocene, much later than the initial human occupation of the continent, and most probably required the entrance of extra morphological diversity from regions outside the continent.

## Supporting Information

S1 Dataset South American Craniometric Data. Craniometric data and contextual information of the South American collections included in this study.
(XLSX)
